# Research on the Mechanical Properties of a Glass Fiber Reinforced Polymer-Steel Combined Truss Structure

**DOI:** 10.1155/2014/309872

**Published:** 2014-08-27

**Authors:** Pengfei Liu, Qilin Zhao, Fei Li, Jinchun Liu, Haosen Chen

**Affiliations:** ^1^Institute of Field Engineering, PLA University of Science & Technology, Nanjing, Jiangsu 210007, China; ^2^Department of Engineering Management, PLA Institute of Logistics Engineering, Chongqing 401331, China; ^3^Institute of Defense Engineering, PLA University of Science & Technology, Nanjing, Jiangsu 210007, China

## Abstract

An assembled plane truss structure used for vehicle loading is designed and manufactured. In the truss, the glass fiber reinforced polymer (GFRP) tube and the steel joint are connected by a new technology featuring a pretightened tooth connection. The detailed description for the rod and node design is introduced in this paper, and a typical truss panel is fabricated. Under natural conditions, the short-term load test and long-term mechanical performance test for one year are performed to analyze its performance and conduct a comparative analysis for a reasonable FEM model. The study shows that the design and fabrication for the node of an assembled truss panel are convenient, safe, and reliable; because of the creep control design of the rods, not only does the short-term structural stiffness meet the design requirement but also the long-term creep deformation tends towards stability. In addition, no significant change is found in the elastic modules, so this structure can be applied in actual engineering. Although the safety factor for the strength of the composite rods is very large, it has a lightweight advantage over the steel truss for the low density of GFRP. In the FEM model, simplifying the node as a hinge connection relatively conforms to the actual status.

## 1. Introduction

GFRP has excellent performance characteristics, such as lightness, high strength, flexibility in design, good antishock performance, and fatigue and corrosion resistance, as well as good protection and concealment function. Moreover, the cost is lower than that of carbon fiber reinforced polymer or basalt fiber reinforced polymer, which is more economically suitable for civil engineering. As a result, GFRP has been applied widely in civil engineering [1–8].

However, GFRP has some obvious defects: a lower elastic modulus and significant creep property. For instance, the tension elastic modulus of pultruded E-glass fiber/913 composite is 42 GPa at room temperature, which is 1/5 of steel's elastic modulus [9]. Meanwhile, the significant creep property alters the endurance performance of the structure and influences the long-term reliability of the structure [10]. Lifshitz and Rotem [11] find that, for a unidirectional glass fiber/polyester composite with a fiber volume fraction of 60%, creep failure occurs over 10^5^ min for under 50% of the initial tensile strength at room temperature. Experiments show that creep is severe when the principal stress direction presents at 45° with longitudinal and latitudinal directions, and the total tensile creep strain of 100 h is about three times the initial strain, which is under 30% of the static strength, and the total creep deflection of 720 h is approximately 5.5 times the initial deflection. Under a stress higher than 50% of the static strength, bending creep failure occurs successively for the samples of 307 glass/polyester composites [12]. Additionally, the connection efficiency of frequently used connection technologies for a composites-adhesive connection, mechanical connection, and bolt-adhesive connection is not generally high, and there is a significant aging problem for the adhesive connection, which restricts the application of GFRPs in civil engineering as the main load-bearing structure. Therefore, the truss structure of GFRP is usually used for pedestrian bridge and roof structures that have a lower bearing capacity and a smaller span. For example, the bolted connection is adopted for a GFRP truss roof structure that is 19 m in length and 4 m in width to bear loads of wind and snow. For a GFRP truss bridge with spans of 12.5 m + 12.5 m and a 16.5 kN self-weight for each span in Switzerland, one span is connected by bolted joints, and the other span is connected by adhesive joints, which bears the pedestrian load [13]. Even if individual organizations developed the GFRP truss highway bridge, the structure is relatively heavier due to the limits of connection technology. Kostopoulos et al. [14] designed and manufactured a GFRP truss bridge, in which the composite members are held together by adhesive bonding and mechanical fastening joints. The bridge has a span of 11.6 m in length and 4 m in width and represents a single traffic lane. The bridge is Class 30 according to DIN 1072 (i.e., load bearing capacity of 300 kN) and weighs approximately 13.5 T, namely, 11.64 kN/m.

In this paper, considering the applications of and the development represented by existing GFRP truss structures in civil engineering as well as the demand for lightweight, modular bridges, for emergency purposes, a GFRP-steel combined truss structure is proposed. It is a vehicular bridge and is suitable for air-transport and manual erection by unskilled workers. It was designed as a modular bridge with a span length of 12 m and consists of 8 truss structural units. To achieve a low weight, GFRP was used for the chord, diagonal, and vertical members based on the consideration of the stiffness requirements of the whole structure. Moreover, a novel connector with a high connection efficiency, known as a pretightened tooth connection (PTTC) structure [15, 16], was employed for the connection of the GFRP material tubes. Based on the proposed design, the weight of the truss panel is a mere 140 kg (40% lighter than the corresponding steel truss), and the bearing capacity of the truss bridge is comparable with the steel truss bridge of the same specifications.

The primary objective of this study was to verify the viability of the proposed GFRP-steel combined truss bridge. A description of the truss structure, including the conceptual design and fabrication is first provided. To investigate the structural performance, a single-layer and single-row assembled simply supported truss bridge with a length of 12 m was erected. The flexural properties of this specimen over both a short period of time and a long period of time were tested according to the principle of midspan moment equivalence. In addition, the corresponding numerical analysis based on two connection types was performed for a reasonable FEM model.

## 2. Conceptual Design and Fabrication for a New Composite Truss

### 2.1. Conceptual Design for a Composite Truss Structure Type

**Figure 1:**
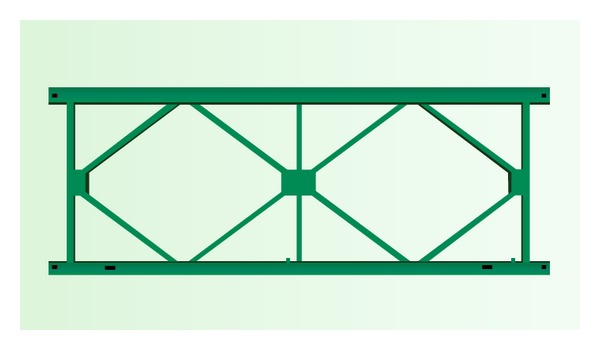
The “321” fabricated highway steel bridge truss panel.

**Figure 2:**
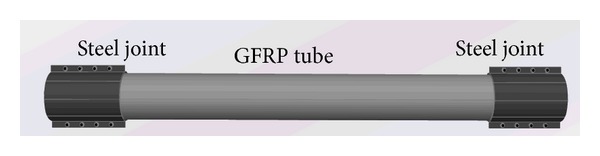
The integrity of the GFRP tube and steel joint.

**Figure 3:**
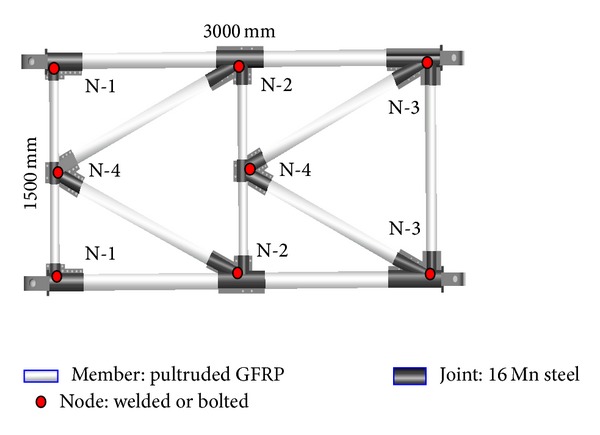
Structural design of the GFRP-steel combined truss.

For convenience in conducting the static load contrast experiment, the external dimension of a typical truss panel is in conformity with the external dimension of the “321” fabricated highway steel bridge truss panel (Figure 1), namely, a length of 3000 mm and a width of 1500 mm. However, for the weight reduction of all nodes, a cross-type truss of the fabricated highway steel bridge is not used for the GFRP-steel combined plane truss, but rather a parallel chord truss with fewer nodes is used. Members of the combined truss are made from a pultruded unidirectional glass fiber reinforced resin matrix composite and the joints are made from 16 Mn steel (Figure 2). They form an integrity that can transfer a load by pretightened tooth connection technology. Composite members of the truss are connected mutually at different type nodes, N-1, N-2, N-3, and N-4, through welding or bolt of steel joints (Figure 3).

### 2.2. Conceptual Design for Members

Common composite pultruded profiles are available in H-shaped, channel, and tubular composite materials. In the design, composite members are connected using pretightened tooth connection technology, and the cross-section of the member is shaped such that it is easy to connect the joints. Thus, tubular composite materials are used. Finally, pultruded glass fiber reinforced isophthalic unsaturated polyester resin composite tubes with a fiber mass fraction that is 61% manufactured by the Nanjing Jinjiuding Composite Company are chosen. Their density is 1.667 g/cm^3^. Mechanical parameters provided by the manufacturer are shown in Table 1.

The geometric size of the member depends on the forced state under actual working conditions. The GFRP-steel combined truss is guaranteed to bear the design load, which is not less than the bearing capacity of an existing “321” fabricated highway steel bridge. Namely, for a 12-meter long, single-row monolayer design, the wheel load is 20 tons in the midspan [17]. The internal force value of this span and load can be easily acquired from the structural mechanism model, as shown in Table 2. The internal force value of the upper and lower chords of the truss is the same, both of which are 200 kN. The upper chord is compressed, and the lower chord is tensed. However, it is considered that the truss can be used for both upper and lower chords, and the compressive strength of composites is obviously less than the tensile strength, so they are all designed as compressive members. To reduce the manufacturing cost and avoid remanufacturing the pultrusion mold, up and down composite tubes with a 104 mm external diameter and 8 mm thickness are selected. For the same reason, composite tubes with a 76 mm external diameter and 8 mm thickness are chosen to serve as the diagonal members and vertical members. Then, with reference to compressive strength, the cross-sectional area of the member, length of the member and elastic modulus of composites, different compressive strengths, and buckling loads for members, as well as safety factors, are determined; see Table 2 for details. Due to the dispersion of GFRP, the minimum safety factor for an actual application is 2 [17]. During the primary design, all of the safety factors of the members are more than 2 [18], while the strength and stability of the members meet the requirement.

### 2.3. Conceptual Design for Joints

**Figure 4:**
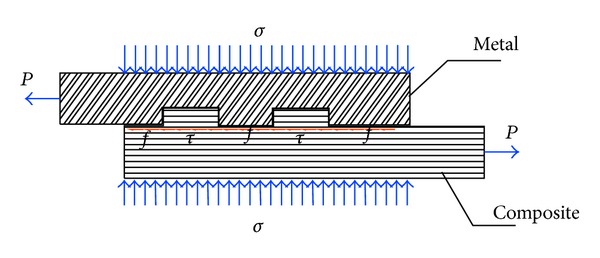
The mechanism diagram of a pretightened tooth connection for composites.

A pretightened tooth connection processes teeth on the composite and transfers the axial force via the shear capability of tooth root, and the interlaminar compressive stress formed by imposing the pretightened force improves the interlaminar shear strength so that the composite can transfer larger load in joints [16] (Figure 4).

**Figure 5:**
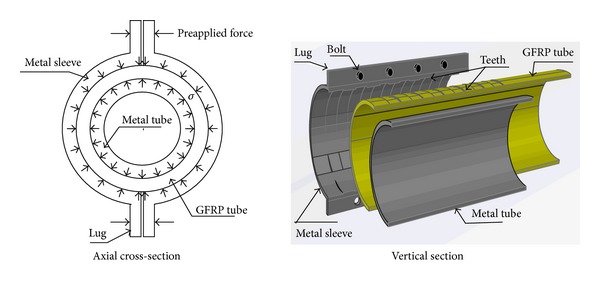
Schematic diagram of the joint.

The circular tube joint of the composite made by pretightened tooth connection technology usually consists of external metallic sleeves with lugs, middle composite tubes, and internal metallic tubes. To fabricate such a joint, a metal tube that has an external diameter that is identical to the inner diameter of a composite tube that has greater stiffness is placed in the composite tube, and, then, the matching teeth are machined on the outer face of the composite tube and the inner face of the metal sleeve. In the last step, the metal tube is assembled along with the composite tube, and a great radial pressure stress is applied on the composite tube as high-strength bolts (Figure 5). The bearing capacity of this connection mode is related not only to the material properties but also to the pretightening force, tooth depth, and tooth length, and tooth quantity.

**Figure 6:**
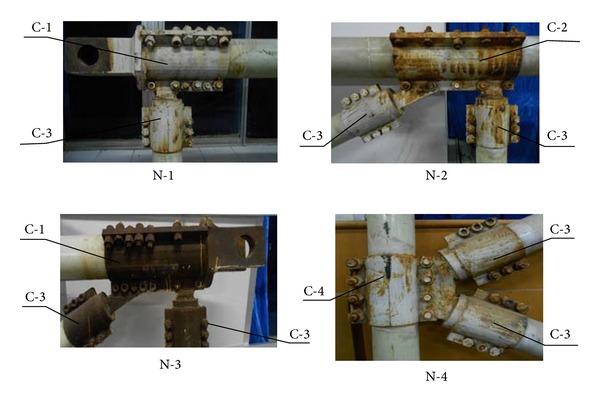
Classification of the joints in the GFRP-steel combined truss.

The design for the member joints of the GFRP-steel combined truss structure mainly involves determining the geometry of the joint's steel sleeve, the tooth quantity, the tooth depth, and the length and value of the preload, based on the requirement for the bearing capacity. The joints can be divided into four categories depending on their locations and stress state (Figure 6): (1) chord end joint C-1: these types of joints are located on the ends of the chord member, are connected to the male or female heads, and serve as the major power transmission parts of the truss structure so they require a large number of teeth and increased preloads; (2) central joints of chord member C-2: these joints connect the vertical members with the diagonals and eliminate the need to pass large loads, thus requiring less teeth and lower preloads; (3) joints at the ends of the diagonal or vertical member C-3: these joints are located on the ends of the diagonals or the vertical members and only require passing the loads imposed on the diagonal or vertical members. As these are not heavy loads, they require less teeth and lower preloads; (4) joints in the middle of vertical member C-4: these nodes mainly work to connect the diagonals without passing heavy loads, thus requiring less teeth and preloads.

The design principles of the joints are as follows: (1) joints can transfer loads of no less than the design loads of the members. (2) Steel is used as little as possible at the joints for the weight reduction of the truss. Therefore, for end joints of the chord members of C-1 that have greater preloads and the joints of C-2 with a larger diameter, the outer steel sleeve needs to be divided into three sections with three pairs of lugs, while the other divided outer steel sleeves require two pairs of lugs. (3) The impact of the welding temperature on the composite tubes is taken into account when joints are fabricated. Based on these principles, in combination with the design method of the composite pretightened tooth connections and adopting safety factors larger than 2, the optimal depth and length of the teeth, the geometry of the joints, and the carrying capacity can be obtained, as shown in Table 3. The inner metal tube and outer steel sleeve are made of 16 Mn steel. There are two types of external diameters of the inner steel tubes, 88 mm and 60 mm, with an 8 mm wall thickness and a length matching the outer sleeves.

### 2.4. Design and Assembly for the Truss Joint

The pretightened tooth connection joints not only can be used for unidirectional extension or multidirectional connections of composite members but also can be easily made into single- or double-lug joints between GFRP-steel combined truss panels and spherical joints. Therefore, they can be widely used in truss bridges, space trusses, and prestressed bridge structures. The joint is designed according to the principle of reasonable construction, reliable connection, and convenient manufacture so that the existing mature weld technology of steel or bolted connections can be used.

**Figure 7:**
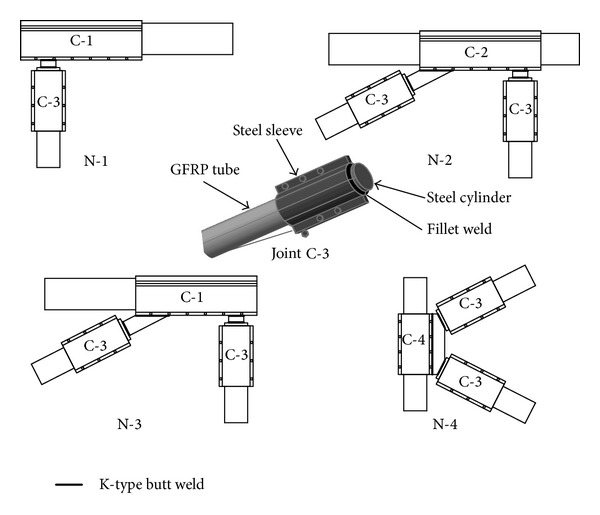
Different types of nodes welded by joint C-3 and other joints.

The rod is simplified to have an axial tension and compression status. While designing the joint by the welding method, the C-3 joint is connected with other types of joints to form different nodes. First, a short steel column with a diameter that is equivalent to the inner diameter of the external sleeve is inserted and an all-around fillet welded at the end of the C-3-type joint. The top surface of this short column is used for welding with other joints. The width of the weld leg *h*_*f*_ = 8 mm, length of weld *l*_*w*_ = 3.14 × 76 mm = 238.64 mm, strength of fillet weld *f*_*f*_^*w*^ = 165 MPa, and the bearing capacity of fillet weld [*N*] = 0.7*h*_*f*_ *l*_*w*_ *f*_*f*_^*w*^ = 220.5 kN is more than the maximum value of 85 kN of the design internal forces for diagonal rods and vertical rods and meets requirement. Second, through a K-type butt weld, the weld at the C-3 diagonal joint, and the lug of chord member joint C-1, the weld at the C-3 diagonal joint and the lug of vertical member C-4, the weld at the C-3 diagonal joint and the lug of chord member joint C-1, and the C-3 joints of the vertical member and diagonal member and the lug of chord member joint C-2 form nodes N-1, N-2, N-3, and N-4 (Figure 7). The butt weld bearing capacity [*N*] is calculated as the most conservative three-level weld joint in tension [19], and the safety factors are uniformly greater than 2 (Table 4).

To the best of our knowledge, this is the first attempt to fabricate this type of truss structure, so there are no norms and standards in this respect. Therefore, the reference is made according to the welding procedures that are used for the existing assembled highway steel truss bridges and composite connection technologies, and these assembly and welding methods are proposed for the structure. This method can effectively reduce the stress associated with the assembly and ensure that all members can be accurately assembled. The method is divided into three steps.

**Step 1 .**

**Figure 8:**
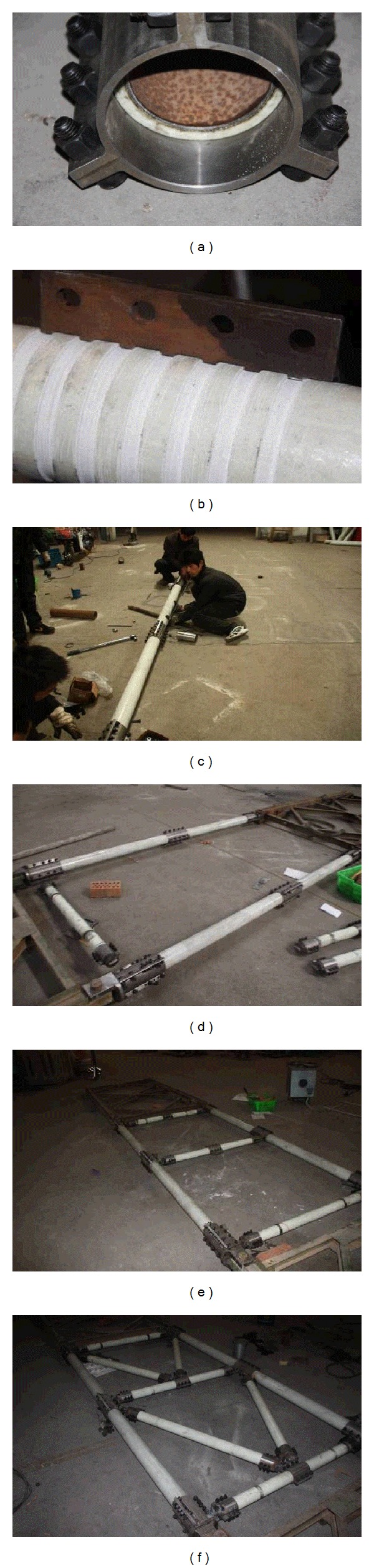
Assembly process of the truss.

Steel joints are prepared, which mainly involves the processing of the inner steel tube, outer steel sleeves, and male and female heads at the ends. To prevent the composite tubes from being damaged by high temperatures while the joints are welded, the steel connection is somewhat longer than what is required for the joints to pass forces (Figure 8(a)). The truss panels are connected by male and female heads that are made specifically for this purpose.

**Step 2 .**

Teeth are machined at the corresponding locations on the composite tubes and assembled with the inner steel tubes and the outer steel sleeve of joints (Figure 8(b)). To ensure assembling accuracy, the threads must be correctly positioned with accurate lengths and depths and perfectly matched with the teeth on the outer steel sleeve. While composite tubes are assembled with steel joints, all of the lugs used for welding must be in the same plane.

**Step 3 .**

Members are assembled. The assembly of members involves welding the steel joints at the ends and in the middle of the members. In the assembly, all of the welded members of the truss panel are in the same plane. To prevent the welding temperature from impacting the composite tubes, cooling measures, such as cold compression using a wet towel, are taken during welding. Chord members are installed first (Figure 8(c)). The specially made male and female heads are connected with the existing assembly highway steel bridge truss on both sides via single-pin connections, before the steel sleeves of the composite chord members are welded with the male and female heads. Then, vertical members are installed (Figure 8(d)). The steel sleeves on the ends of the vertical members are welded to the lugs of the chord members. The center line of the vertical member must coincide with the center of the lug of the chord member. Meanwhile, the lugs of the three vertical members remain in the same plane to ensure that the diagonals are in the same plane while they are welded (Figure 8(e)). Finally, the diagonals are installed (Figure 8(f)). The steel sleeves on the ends of the diagonals are welded to the lugs of the steel sleeves in the middle of the vertical members. The center line of the diagonal must coincide with the lug of the vertical member.

The assembled composite truss panel weighs 160 kg, which is 40% lighter than the 270 kg steel truss panel of “321” assembled highway steel bridges.

## 3. Mechanical Performance Test and Analysis of a GFRP-Steel Combined Truss Structure

### 3.1. Structure Test Scheme

#### 3.1.1. Short-Term Loading and Test Scheme

**Figure 9:**
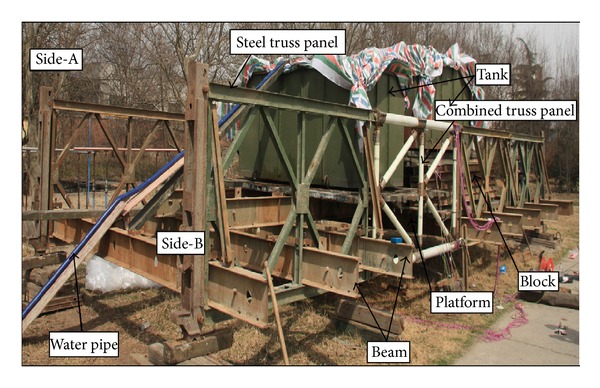
Static live loading mode of the structure.

To verify the reliability of the combined truss panel, a single-layer and single-row assembled simply supported highway steel bridge with a length of 12 m is erected. Therein, Side-A consists of four steel truss panels and Side-B consists of three steel truss panels and one combined truss panel in the midspan. According to the principle of midspan moment equivalence, the mode that the steel members and steel tanks are stockpiled on the platform of deck slabs and the water is injected into the box shall be used to simulate a 20 t wheel load. The load is transferred to the main truss through four beams in the midspan (Figure 9).

**Figure 10:**
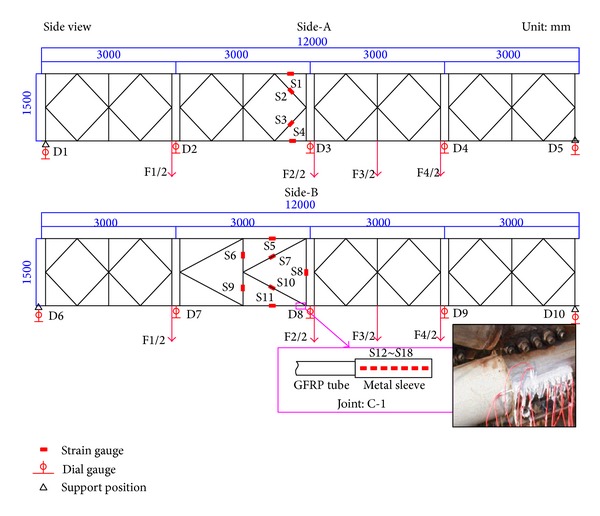
Static live loading position and sensor arrangement.

The deflection of the bridge and the stress of the key rods and joints are tested during loading. Dial indicators with manual readings are set in the location of the midspan, *L*/4, and support position of each side. Five test points are set at each side, totaling 10 test points on both sides. The numbering is from D1 to D10. The strain gages are set on the upper chord, lower chord, and diagonal members of the truss in midspan of Side-A. The numbering is from S1 to S4, and the internal force of steel rod is tested. The strain gages are set on the upper chord, lower chord, and vertical and diagonal members of the combined truss in the midspan of Side-B. Its numbering is from S5 to S11, and the state of composite rods shall be tested. In addition, the strain gages are set on joint C-1 at the end of the lower chord of the combined truss panel. The numbering is from S12 to S18, and the stress state is tested (Figure 10).

The equivalent bending moment starts at 0 and progressively increases to 600 kN*·*m under 6-step loadings. The bearing reactions distributed to the main truss by transverse beams during step loading is maintained for 2 mins after each step loading for the sufficient development of deformation, and structural deflections and stress are collected, see Table 5. The truss is monitored in real time during loading; in cases of the obvious bending of the composite rods, obvious looseness of the steel joints and rods, or notable broken soundness of the material, the loading is stopped immediately.

#### 3.1.2. Test Scheme for Long-Term Loading

The long-term performance of the truss affects the safety of the truss structure. The long-term performance indexes consist of the reduction rate of the existing bearing capacity of the truss structure, deflection of the truss structure, and corrosion and damage degree of the truss structure. In view of the more obvious creep property of GFRP, long-term deflection monitoring and research on stiffness changes one year later are conducted for the truss structure.

The load for the long-term deflection test is the sum of the self-weight of the truss structure and the steel weight on the loading platform. The midspan equivalent moment is 300 kN*·*m; that is, the upper chord of composite truss bears 42 MPa, of which 13% of the stress is the ultimate strength and is lower than the limitation of 30% for long-term creep control stress [20, 21]. The long-term deflection change is tested with balance level.

**Figure 11:**
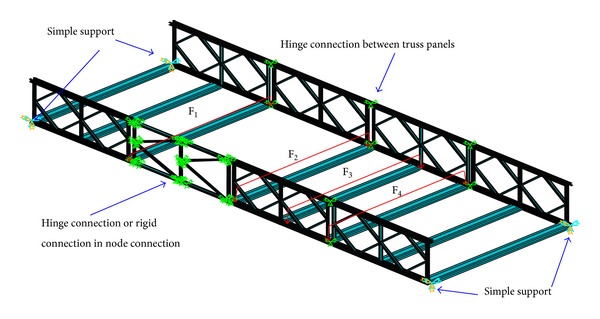
The finite element method model.

One test point per 3 m is arranged under the lower chord of the truss on both sides and 5 are arranged on each side, totaling 10, the position of which is identical to that of the dial indicators in Figure 11. The survey has been performed continuously for over one year from March 2011 to April 2012, with a frequency of once per 15 days during the first three months, then once per month during the second three months, and, finally, once per half-year after six months. The temperatures were between −8°C and 39°C, with an average temperature of 16.4°C and an average relative humidity of 76%.

After one year, in April 2012, the truss is added with the step equivalent bending moment of static live loads 60 kN*·*m, 120 kN*·*m, and 240 kN*·*m according to the short-term performance test method from one year earlier. The total added load is 540 kN*·*m. The change of one side deflection for the GFRP-steel combined truss structure is tested during the loading; the test point distribution is shown in Figure 10.

### 3.2. Structural Mechanical Property Analysis under a Short-Term Load

#### 3.2.1. FEM Model

The FEM analysis software ANSYS 12.1 developed by ANSYS, Inc., in Pennsylvania, USA, is used to simulate the stress and deformation under midspan equivalent bending moments of 120 kN*·*m, 240 kN*·*m, 300 kN*·*m, 360 kN*·*m, 480 kN*·*m, and 600 kN*·*m that are generated by loadings *F*_1_, *F*_2_, *F*_3_, and *F*_4_ on four beams in mi-span scattered across both sides in the experiment. In the case of two simple supported ends, the deflection, axial stress, and axial strain under a load with various levels can be obtained (Figure 11).

Regarding the steel truss structure, the Mohr formula is usually adopted for a theoretical deflection calculation to simplify the node to hinge connection, and only the axial tension and compression stress are considered. Due to large steel modulus, disregarding such secondary stress, as the shear stress in the Mohr Formula is feasible, but for the resin matrix composite with a low modulus, it is unknown whether the effect should be considered in the new truss structure. Therefore, BEAM188 element modeling is used in consideration of the above situation for increased accuracy, with values that are closer to the actual value. Please refer to Tables 1 and 2 for the parameters and cross-section forms of the composite truss. For the GFRP-steel combined truss structure, the performance in nodes between composite and steel is different. Because it is still unknown whether they can be simplified through a calculation, when building models, analogue experiments for both the hinge connection and rigid connection in the node connection are conducted to obtain the result that determines which one is more suitable.

#### 3.2.2. Analysis of the Structural Stiffness Performance

The vertical stiffness of a truss is related to the cross-section, elastic modulus, and structural distribution, which is inversely proportional to the deflection. The elastic deflection generated by the static live load of the truss bridge is the main control value for verifying the vertical stiffness [22]. As a result, the calculated value and experimental value of deflection for this truss bridge are analyzed.

**Figure 12:**
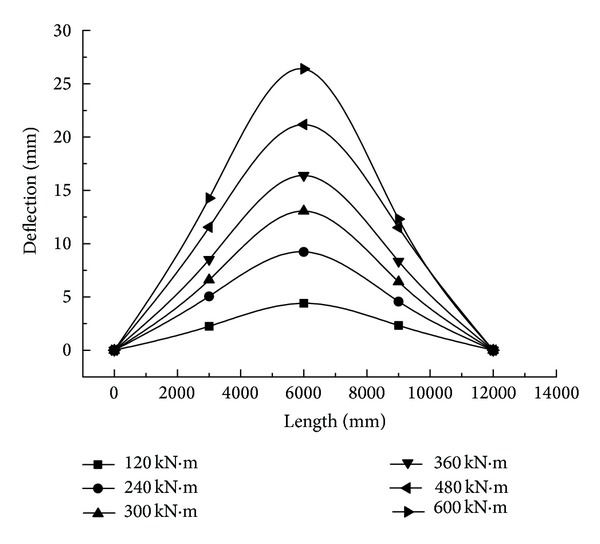
Deflection of Side-B with GFRP-steel combined truss.

**Figure 13:**
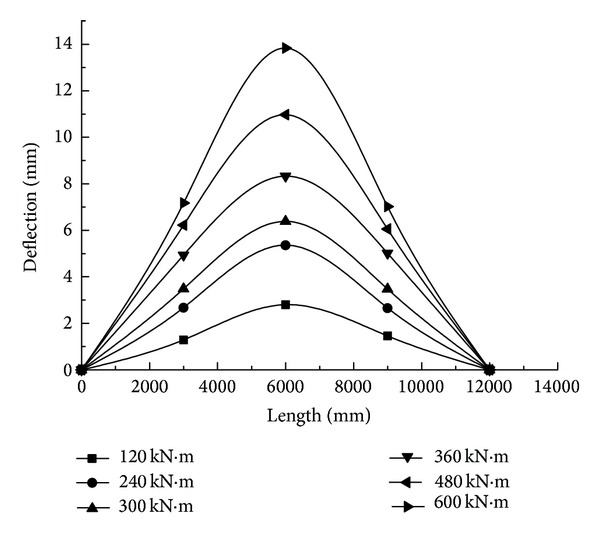
Deflection of Side-A.

**Figure 14:**
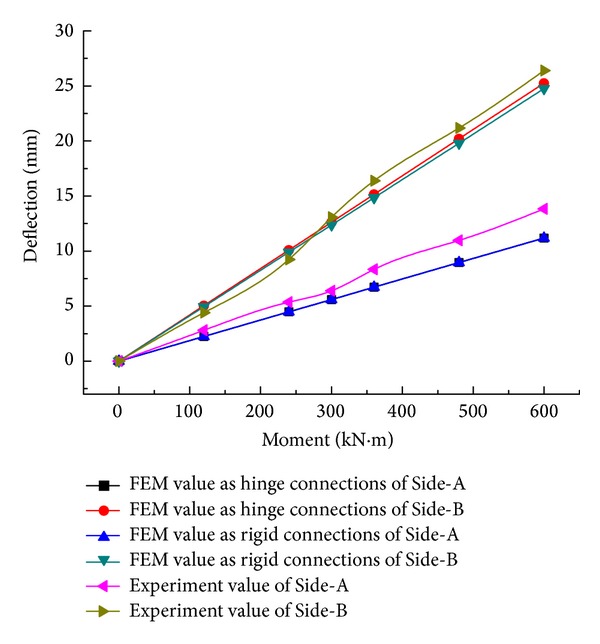
Finite element calculated values and the experimental values of deflection in the midspan for both sides.

Under different step loadings, deflections on both sides of the truss structure that change in the longitudinal direction are indicated in Figures 12 and 13. It turns out that, in either Side-A or Side-B, with the combined truss, in each phase of loading, the deformation of the truss is the same as well as the maximum deflection on both sides of truss structure in the midspan. Owing to the stiffness of a GFRP-steel combined truss structure being lower than that of a steel truss, the deflection on Side-B is distinctly larger than that on Side-A (Figure 14). Under operating conditions of maximum loading, the displacement on Side-B is 1.67 times that of Side-A, which proves that the stiffness of glass fiber structure is most probably the control factor of this structure.

Meanwhile, after comparing the maximum experimental value of deflection on both sides of the truss structure in the midspan with the FEM value, the difference is distinct but tends to be of a stable value. This is because the experimental value of deflection for fabricated highway steel bridges includes elastic deflection and inelastic deflection; nevertheless, the calculated FEM value is only the elastic deflection under a static live load. The elastic deflection is the elastic deformation produced under the effect of dead weight and the static live loading of the truss structure. The inelastic deflection is the inelastic deformation of the structure caused by relative displacement because of the design clearance between the pins and the holes of pin joints in the truss [22]. Generally, due to the friction between the pin and the hole, the inelastic deflection will gradually develop to the maximum, with an increase in the static live load. Therefore, the difference between the experimental value and the theoretical value under the loading is the residual inelastic deflection.

From Figure 14, for Side-A, the difference in the calculated finite element values between the rigid connection and hinge connection, both of which are transferred from the internal nodes of the steel truss panel, is slim, whereas, the value for the hinge connection is larger than the rigid connection when transferred from the internal nodes of the combined truss panel in Side-B, which approaches the experimental value. Accordingly, converting the nodes inside the combined truss panel to the hinge connections is more practical and simplifying, as the hinged connection is suitable because the elastic modulus of the composite is lower, and when one rod is rotating, not only does the opposite joint follow suit easily but also the restrain from the other rods is also relatively slim.

To determine whether the deflection for the simply supported truss bridge with a 12 m span, which uses combined truss panels, meets the specified limitation under a 20 t wheel load, the finite element ANSYS software is utilized to build the finite element model, and a 200 kN static live load is imposed on midspan in this model. The maximum midspan deflection calculated is 66.003 mm. The design specifications on the simple truss steel bridge for emergency use provide that when the span *L* < 50 m, the vertical elastic deflection generated by the static live load is *f* ≤ *L*/150. In this case, it is 80 mm. This indicates that the new composite truss bridge satisfies the stiffness requirements.

#### 3.2.3. Analysis of the Structural Strength Performance

**Figure 15:**
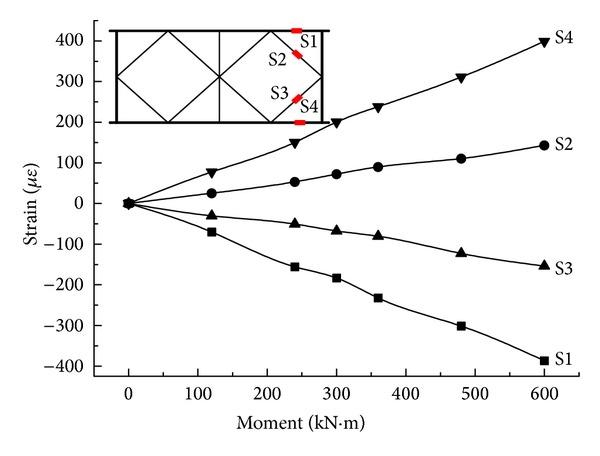
The relationship between the equivalent bending moments and experimental values of strain for Side-A.

**Figure 16:**
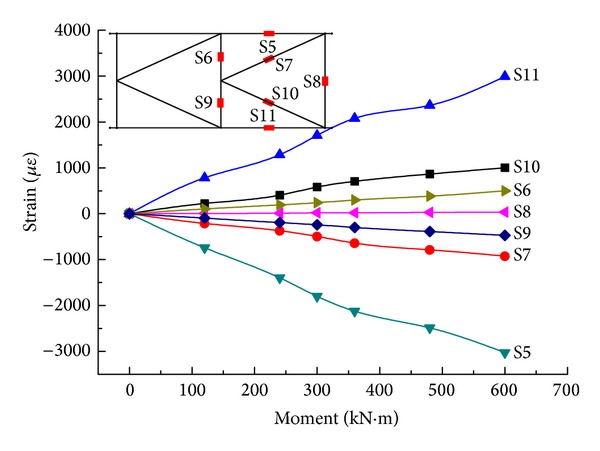
The relationship between the equivalent bending moments and experimental values of strain for Side-B.

By step loading, the relationship between the equivalent bending moment in the midspan on both sides and strain of the rods is determined, as shown in Figures 15 and 16.

From Figures 15 and 16, in either the steel truss member or the composite rod, the overall bending moment-average longitudinal strain changes in a linear form. All of the strain values for the upper chords are negative, while all of the strain values for the lower chords are positive, but, basically, their absolute values are the same. The same is true for the diagonal rod and vertical rod. This is in agreement with the theoretical analysis. At the same time, by the combination of the elastic modulus with a cross-sectional area of the rod, the experimental internal force value and FEM value (Table 6) for combined truss rods can be obtained at a value under 600 kN*·*m. The FEM value is well matched with the experimental value; therefore, the model is accurate. The experimental value and FEM value are both less than the design value, while the rod strength meets the requirement and the rods are provided with large safety factors.

**Figure 17:**
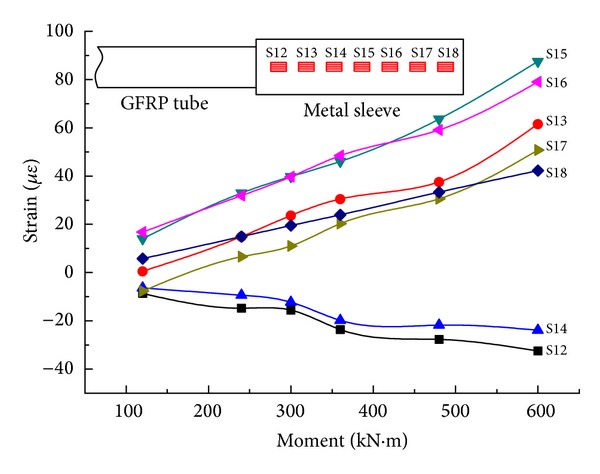
The relationship between the average longitudinal strain and bending moment of steel joint C-1 on the lower chord of a GFRP-steel combined truss.

The main concern of the experiment is that joint C-1 pulls on the lower chord without determining the relative sliding between the composite tube and metal sleeve. Refer to Figure 17 for the relationship between the average longitudinal strain and the bending moment of steel joint C-1 on the lower chord of GFRP-steel combined truss structure. When the lower chord is subjected to pull, the test point should be positive, in theory, but it appears that the positive and negative strains alternate in the joint, of which the negative strain is caused by the eccentric bending moment that is produced by the load on the internal teeth of steel sleeve. Regarding various strains in different test points and loads, the changing rule is more or less constant. The average longitudinal strain on the surface of the steel joint becomes larger linearly with an increase in the bending moment. Under the maximum bending moment of 600 kN*·*m, the maximum longitudinal strain is  87.5 *με*, which is less than the yield strain 1668 *με* of 16 Mn steel and without relative sliding. It proves that the pretightened tooth connection technology of the composite is reliable under the design load.

### 3.3. Analysis of the Structural Mechanical Property under a Long-Term Load

The study of the mechanical property under long-term loading is based on the creep effect of the composite to the truss structure, namely, aiming at the long-term deflection and stiffness of the combined structure.

**Figure 18:**
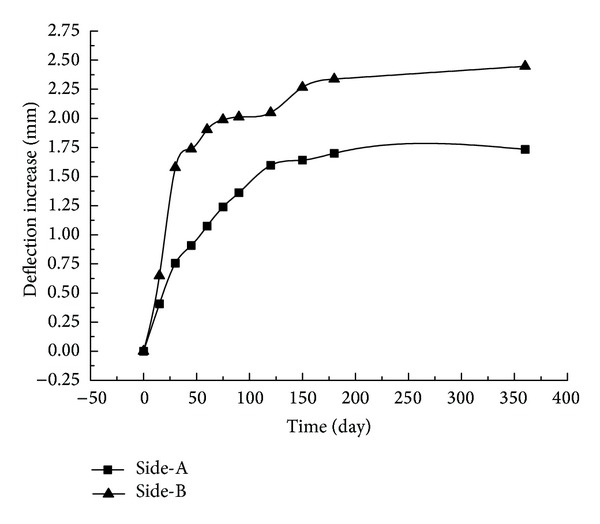
The relationship between the deflection increment of the midspan on both sides of the truss structure and time.

Figure 18 shows the relationship between the deflection increment of the midspan on both sides of the truss structure and time.

From Figure 18, the deflection of Side-A of the truss structure increases with time. After 360 days, the total change is 1.73 mm, accounting for 27% of the short-term experimental value of 6.381 mm under 300 kN*·*m, and the change rate for the deflection increment tends to be stable over time. The deflection on Side-B increases with time, and, after 360 days, the total change is 2.45 mm, accounting for 19% of the short-term experimental value of 13.064 mm under 300 kN*·*m; the change rate for the deflection increment tends to be stable over time. The deflection change rate of Side-B with the GFRP-steel combined truss is 42% larger than that of Side-A without the GFRP-steel combined truss, not exceeding 3 mm in total, which verifies that the long-term performance of the GFRP-steel combined truss is very stable.

**Figure 19:**
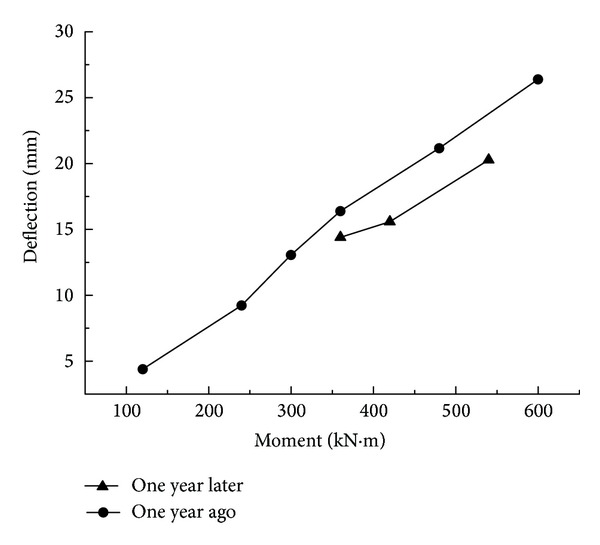
The relationship curve between midspan deflection and the bending moment for Side-B during one year.

Composite materials are easily affected by time, temperature, humidity, and other factors, which cause a change in mechanical properties. One important manifestation is the relationship of elastic deflection increments with bending moment increments. From Figure 19, the relationship curve between the midspan deflection and the bending moment from one year ago basically parallels the relationship curve between the midspan deflection and the bending moment from one year later. The change trend is consistent, namely, the overall stiffness of Side-B with the combined truss panel that remains unchanged shows that the long-term exposition property of the GFRP-steel composite truss structure is stable under natural conditions.

The loading in the test increased stepwise up to 540 kN*·*m. As there is no relative sliding at the connecting part during loading, the pretightened tooth connection joint is reliable.

The analysis shows that the GFRP-steel combined truss has a stable performance and is suitable to use for temporary emergency bridges.

## 4. Conclusion

In this study, a GFRP-steel combined truss is put forward to make a typical truss panel. Furthermore, load tests and analyses are conducted in a 12 m span with a 20 t equivalent wheel load. The research shows the following.

(i) The ends of composite rods are fabricated with steel joints by way of pretightened tooth connections. The mature weld technology is convenient for the design and manufacture of various nodes of the composite combined truss, and the experiment proves the reliability of the transmission of a load.

(ii) The structural deflection of the designed combined structure with a 12 m span under a 20 T equivalent wheel load is 66.003 mm less than the standard specified *L*/150, namely, 80 mm. The short-term stiffness meets the requirement. While the long-term stress state of the GFRP rod is less than 30% of the design strength, the structural long-term creep deformation tends to be stable over 3-4 months and the long-term deformation is 1.19 times the previous elastic deformation.

(iii) One year later, the actual test for the stiffness of the combined truss structure is conducted. Compared with the stiffness at the beginning of its erection, the stiffness of the truss structure does not decrease, which indicates that the elastic modulus of the glass fiber composite material does not decrease significantly under natural conditions and no relative sliding occurred at the pretightened tooth connecting joints; therefore, the structural stiffness is reliable.

(iv) In the span and design load, the weight decreases by 40%, with the steel truss panel weighing 270 kg and the GFRP-steel combined truss panel weighing 160 kg, which proves that the combined truss has an advantage of being lightweight.

In the future, the fatigue performance of the combined structure will be investigated.

## Figures and Tables

**Table 1:** Mechanical properties of glass fiber reinforced isophthalic unsaturated polyester resin composite tubes at 23°C and 50% relative humidity.

Elastic modulus (GPa)			Shear modulus (GPa)			Strength (MPa)		
*E*_1_	*E*_2_	*E*_3_	*G*_12_	*G*_13_	*G*_23_	*σ*_*t*_	*σ*_*c*_	*τ*_12_
25	3	3	1.2	1.2	9.6	1324	330	35

“1” indicates the fiber direction; “2” and “3” indicate directions that are perpendicular to the fiber. Direction 3 is orthogonal with direction 2. “*t*” indicates tension. “*c*” indicates compression.

**Table 2:** Size and performance of the composite truss members.

Location	*L* (mm) × number	ED (mm)	*T* (mm)	IFE	DF	IFSF	CFB	SF
Chord	2800 × 2	104	8	200	250	796	718	2.9
Vertical	1292 × 3	76	8	25	37.5	564	302	8
Diagonal	1500 × 4	76	8	56	85	564	224	2.6

*L*: length; ED: external diameter; *T*: thickness; IFE: internal force under a 20 t external load; DF: designed internal force under external load and dead weight; IFSF: internal force for strength failure; CFB: critical internal force for elastic bulking; SF: safety factor.

**Table 3:** Size and bearing capacity of the joints.

Type	NT	DT (mm)	WT (mm)	*P* (kN)	TT (mm)	TS (mm)	PN	CC (kN)	DF (kN)
C-1	5	2	12	60	8	8	3	600	250
C-2	3	2	12	30	8	8	3	300	85
C-3	3	2	12	30	8	8	2	300	37.5
C-4	3	2	12	30	8	8	2	300	85

NT: number of teeth; DT: depth of tooth; WT: width of tooth; *P*: preload; TT: thickness of the inner steel tube; TS: thickness of the outer sleeve; PN: pair number of lugs; CC: carrying capacity; DF: designed internal force.

**Table 4:** Size and bearing capacity of the butt weld.

Group	*t* (mm)	*l*_*w*_ (mm)	*f*_*t*_^*w*^ (MPa)	[*N*] (kN)	DF (kN)	SF
Diagonals C-3 and C-2 or C-1	16	70	250	280	85	3.3
Diagonals C-3 and C-4	16	45	250	180	85	2.1
Vertical members C-3 and C-2 or C-1	16	40	250	160	37.5	6.7

*t*: weld thickness; *l*_*w*_: length of weld leg; *f*_*t*_^*w*^: tensile strength of butt weld; [*N*]: bearing capacity of fillet weld; DF: designed internal force; SF: safety factor.

**Table 5:** Step loading in the experiment.

EBM (kN*·*m)	120	240	300	360	480	600
*F*_1_ (kN)	8.9	17.8	22.3	26.7	35.6	44.5
*F*_2_ (kN)	20.7	41.5	51.9	62.2	83.0	103.7
*F*_3_ (kN)	11.8	23.7	29.6	35.5	47.3	59.2
*F*_4_ (kN)	11.8	23.7	29.6	35.5	47.3	59.2

EBM: equivalent bending moment.

**Table 6:** Contrast experimental value with the FEM value of composite rods under 600 kN*·*m.

Member	S5	S6	S7	S8	S9	S10	S11
IFD (kN)	200	25	56	25	25	56	200
EV (kN)	−182.4	19.8	−42.5	1.3	−19.9	42.8	180.3
FEM (kN)	−190.8	20.5	−43.9	1.4	−20.7	43.9	183.3
Deviation	4.6%	3.5%	3.3%	7.7%	4%	2.6%	1.7%

IFD: internal force value for 20 t designed loading; EV: experimental value; deviation: deviation = |(EV − FEM)/EV| × 100%.

## References

[1] Gilby J (1998). Pultrusion provides roof solution. *Reinforced Plastics*.

[2] Gonilha JA, Correia JR, Branco FA (2013). Creep response of GFRP-concrete hybrid structures: application to a footbridge prototype. *Composites B: Engineering*.

[3] Gonilha JA, Correia JR, Branco FA (2013). Dynamic response under pedestrian load of a GFRP-SFRSCC hybrid footbridge prototype: experimental tests and numerical simulation. *Composite Structures*.

[4] Peirick L (2011). *Connection development and in-plane characteristics of innovative 3-D GFRP composite structural insulated panels for sustainable civil engineering infrastructure applications [M.S. thesis]*.

[5] Santos Neto ABDS, La Rovere HL (2010). Composite concrete/GFRP slabs for footbridge deck systems. *Composite Structures*.

[6] Correia J, Cabral-Fonseca S, Branco F, Ferreira J, Eusébio MI, Rodrigues MP Durability of glass fibre reinforced polyester (GFRP) pultruded profiles used in civil engineering applications.

[7] Jeong J, Lee Y, Park K, Hwang Y (2007). Field and laboratory performance of a rectangular shaped glass fiber reinforced polymer deck. *Composite Structures*.

[8] Mendes PJD, Barros JAO, Sena-Cruz JM, Taheri M (2011). Development of a pedestrian bridge with GFRP profiles and fiber reinforced self-compacting concrete deck. *Composite Structures*.

[9] Wang T (2012). *Finite element analysis for simulate composite wing of Su-27 and higher-order theory [M.S. thesis]*.

[10] Yang T (1992). *Viscoelastic Mechanics*.

[11] Lifshitz JM, Rotem A (1970). Time-dependent longitudinal strength of unidirectional fibrous composites. *Fibre Science and Technology*.

[12] Shanghai FRP Research Laboratory (1980). *FRP Structure Design*.

[13] Bai Y, Keller T, Vall EET, Smith ST Dynamic behavior of an all-FRP pedestrian bridge.

[14] Kostopoulos V, Markopoulos YP, Vlachos DE (2005). Design and construction of a vehicular bridge made of glass/polyester pultruded box beams. *Plastics, Rubber and Composites*.

[15] Li F (2012). *Research on new type of composite connection and application in the truss [Ph.D. thesis]*.

[16] Ma Y, Zhao Q (2011). Analysis of the bonded-bolted hybrid composite joints' carrying capacity. *Acta Materiae Compositae Sinica*.

[17] Huang S, Liu M (2001). *Multi-Purpose Manual of Assembled Highway Steel Bridge*.

[18] Sedlacek G, Trumpf H, Castrischer U (2004). Development of a light-weight emergency bridge. *Structural Engineering International*.

[19] Cao S (2002). *The Principle of Engineering Structure Design*.

[20] Scott DW, Zureick A (1998). Compression creep of a pultruded e-glass/vinylester composite. *Composites Science and Technology*.

[21] Batra S (2009). *Creep rupture and life prediction of polymer composites [M.S. thesis]*.

[22] Hu Y (2001). *Military Bridges*.

